# Multiple hospitalisations towards the end of life among patients with serious mental illness: A retrospective cohort study in England, UK

**DOI:** 10.1192/j.eurpsy.2021.1069

**Published:** 2021-08-13

**Authors:** E. Chukwusa, R. Wilson, F. Gaughran, G. Wei

**Affiliations:** 1 Cicely Saunders Institute Of Palliative Care, Policy & Rehabilitation, kings college London, London, United Kingdom; 2 Dept Of Psychosis Studies, Institute of Psychiatry, Kings College London, London, United Kingdom

**Keywords:** Multiple Hospitalisation, end-of-life, Palliative care, Serious Mental Illness

## Abstract

**Introduction:**

Multiple hospitalisations towards the end of life is an indicator of poor-quality care. Understanding the characteristics of patients who experience hospitalisations at the end-of-life and how they vary is important for improved care planning.

**Objectives:**

To describe socio-demographic and clinical characteristics of patients diagnosed with serious mental illness who experienced multiple hospitalisations in the last 90 days of life.

**Methods:**

Data for all adult patients with a diagnosis of serious mental illness who died in 2018-2019 in England, UK were extracted from the National Mental Health Services Data Set linked with Hospital Episode Statistics and death registry data. Variables of interest included age, gender, marital status, underlying and contributory cause of death, ethnicity, place of death, deprivation status, urban-rural indicator, and patient’s region of residence. The number of hospitalisations and patient’s sociodemographic & clinical were described using descriptive statistics and percentages, respectively.

**Results:**

Of the 45924 patients, 38.1% (n=17505, Male=42.9%, Female=57.1%, Mean age:78.4) had at least one hospitalisation in the last 90 days of life. The median number of hospitalisations was 2(StdDev:1.64, Minimum=1,Maximum=23). Most of those hospitalised (n=11808, 67.5%), died in a health care establishment (e.g. Hospital or hospice). There were marked geographic differences in the proportions of hospitalisations.The North West region of England recorded the most hospitalisations (n= 2906,16.6%), compared to other regions.

**Conclusions:**

Further analysis is needed to understand factors independently associated with hospitalisations in people with serious mental illness. Funding: This project is supported by the National Institute for Health Research (NIHR) Applied Research Collaborations (ARC) South London.

